# Apalutamide-induced life-threatening dermatologic toxicity: Clinical histopathological correlations and salvage therapies in four cases: Case report series

**DOI:** 10.1097/MD.0000000000045425

**Published:** 2025-11-14

**Authors:** Yuanyuan Li, Yi Cao, Lei Wei, Jingyi Zhao, Maocan Tao, Xiaoqing Zhao

**Affiliations:** a Department of Dermatology, The First Affiliated Hospital of Zhejiang Chinese Medical University (Zhejiang Province Hospital of Chinese Medicine), Hangzhou China; b Department of Dermatology, Ruijin Hospital, Shanghai Jiaotong University School of Medicine, Shanghai, China.

**Keywords:** adverse events, apalutamide, case report, prostate cancer

## Abstract

**Rationale:**

Apalutamide is a novel next-generation oral antiandrogen receptor targeting agent for castration-resistant prostate cancer. However, it is associated with skin adverse events (AEs), which have been reported in clinical trials but are not fully understood due to limited clinical data. This case report explored the clinical presentation and management of apalutamide-associated skin events through 4 representative cases.

**Patient concerns:**

Four male patients with prostate cancer developed various skin rashes after taking apalutamide. The rashes were characterized by different manifestations, including erythema, itching, flaking, swelling, and even mucosal erosion, which significantly affected the patients’ quality of life and required prompt intervention.

**Diagnoses:**

Based on the clinical presentation and histopathological examination of the skin, the diagnoses included eczema and severe drug rashes such as toxic epidermal necrolysis and Stevens–Johnson syndrome. The incubation periods of the rashes ranged from 1 week to 2 months, and some patients also exhibited other symptoms like elevated white blood cell count, eosinophilia, increased C-reactive protein and lactate dehydrogenase levels, lymph node enlargement, fever, liver damage, and nail dystrophy.

**Interventions:**

All patients discontinued the use of apalutamide. They were treated with oral antihistamines and topical glucocorticoid ointment. Three cases received systemic glucocorticoid therapy, and 1 case was administered Duplizumab. The treatment strategies were adjusted according to the severity and progression of the rashes.

**Outcomes:**

The rashes gradually improved after the discontinuation of apalutamide and the initiation of appropriate treatment. In one case, the rash reappeared after the patient self-administered apalutamide again, but it subsided on its own after discontinuation of the drug. The symptoms of the other patients were well controlled, and no significant complications were observed during the follow-up period.

**Lessons:**

This case report highlights the importance of recognizing and managing apalutamide-associated skin AEs. The development of these AEs appears to be dose-dependent, and early discontinuation or dose reduction of the drug is crucial for controlling the symptoms. A multidisciplinary approach involving oncologists and dermatologists is recommended to optimize the treatment strategy and maintain the patients’ quality of life.

## 1. Introduction

Apalutamide, a novel next-generation oral antiandrogen receptor targeting agent for castration-resistant prostate cancer. Apalutamide was approved by the U.S. Food and Drug Administration in 2018 for the treatment of nonmetastatic castration-resistant prostate cancer.^[1]^ Food and Drug Administration approved apalutamide for the treatment of patients with metastatic castration sensitive prostate cancer on September 17, 2019. An apalutamide skin rash is an adverse event (AE). According to Selection of Patients with Advanced Resistant Tumors Androgen Receptor-directed Therapy Using Apalutamide for Nonmetastatic Castration-Resistant Prostate Cancer and Targeted Intervention with Apalutamide for the Treatment of Metastatic Androgen-Sensitive Prostate Cancer trials, rash incidence was 23.8% and 27.1% in apalutamide versus 5.5% and 8.5% in placebo groups, respectively.^[2]^ Apalutamide-associated skin adverse events are more common in the global population. However, limited clinical data have hampered further understanding. We present 4 representative cases of apalutamide-associated skin rash that was evaluated symptomatically and histologically. The patients in the 4 case reports has provided informed consent for the publication of this case report. This consent ensures compliance with the CARE guidelines, which mandate the inclusion of a patient consent statement. This study was approved by the Institutional Review Board of the The First Affiliated Hospital of Zhejiang Chinese Medical University. The patients signed the informed consents to approve the use of their information.

## 2. Cases presentation

### 2.1. Case 1

**Figure 1.**
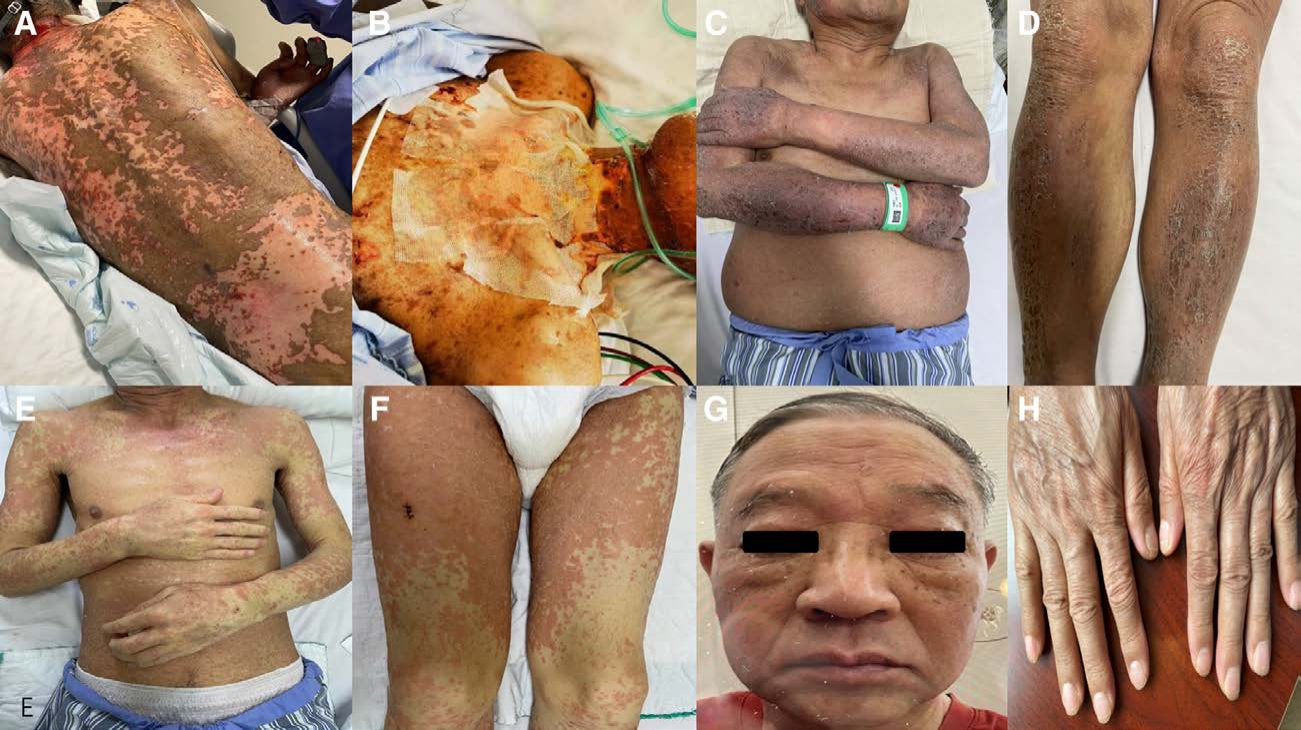
Clinical manifestations of apalutamide-associated skin adverse events. (A) Generalized erythema and papules with a heavy burden on the posterior back, indicative of a severe drug rash. (B) Extensive exudation and desquamation affecting the lower limbs, with evidence of treatment with topical dressings. (C) Close-up view of the abdomen showing erythematous patches and mild desquamation. (D) Edematous and erythematous changes on the arm, demonstrating the systemic nature of the skin reaction. (E) Generalized erythema and flakiness on the chest, with a symmetrical distribution pattern. (F) Erythematous papules on the thighs, some of which are coalescing into larger plaques. (G) Facial involvement with erythema and mild edema, affecting the patient’s appearance and comfort. (H) Palmar erythema and desquamation, highlighting the involvement of acral surfaces in the skin reaction.

A 67-year-old male diagnosed with prostate cancer with bone metastasis in July 2021, was treated with apalutamide and norethindrone. The standardized dose, 240 mg/d, was taken once-daily. After 2 months, erythema of hands and feet with itching appeared, which was not treated. 1 month later, generalized erythrodermic changes appeared, with swelling of hands and face, accompanied by exudation (Fig. 1A, B). Significant increase in eosinophil count, elevated white blood cell count, increased liver enzyme levels, and accompanied by persistent high fever symptoms for 1 week and bilateral axillary lymph node enlargement were observed. Apalutamide and norethindrone were discontinued and methylprednisolone systemic intravenous therapy was given in the outside hospital, but mucosal erosion of the oral cavity and urethral orifice appeared during the treatment, and the dose of hormone was increased to a maximum of methylprednisolone 160 mg bid. Pneumonia and hypoxemia appeared during the treatment, and the pathogenicity examination was perfected in conjunction with the anti-infective therapy. Symptoms were still poorly controlled. After inviting Ruijin Hospital dermatology department for consultation, added intravenous gamma globulin (5 g/d) and plasma replacement (200 mL/time) alternating treatment, the skin lesions were still progressing. The patient was later transferred to Ruijin Hospital dermatology department for treatment on December 6, 2021.

Past history: history of hypertension, regularly taking felodipine; Prostate aspiration biopsy + prostate 125 iodine particle implantation under general anesthesia on July 22, 2021.

Specialized examination: scattered erythema with exudation on the head, face and ears, and a large number of black scabs around the mouth and lips; scattered erythema and vesicles with exudation on the trunk, with a heavier burden on the posterior back; scattered vesicles on the dorsum of both hands and the dorsum of both feet, and vesicles were seen on the urethral orifice and the mucous membranes of the oral cavity.

**Figure 2.**
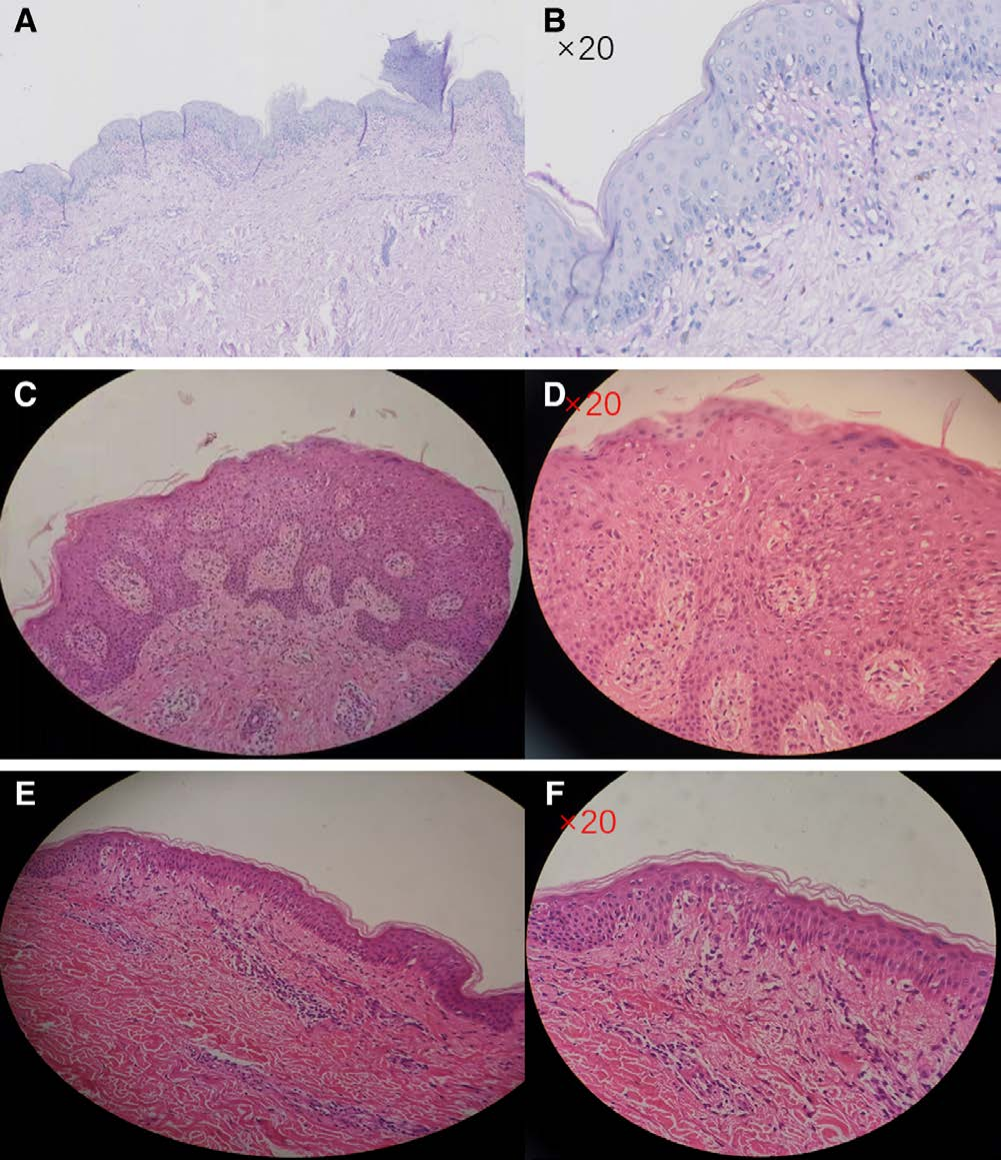
Histopathological features of apalutamide-associated skin adverse events. (A) Epidermal hyperkeratosis with focal hyperkeratosis, indicative of a severe drug-induced skin reaction (H&E stain, 20× magnification). (B) Scattered necrotic keratinocytes in the stratum spinosum, along with vacuolar degeneration in the basal layer, suggesting toxic epidermal necrolysis (H&E stain, 20× magnification). (C) Infiltration of lymphocytes and histiocytes in the superficial dermal layer around blood vessels, consistent with an inflammatory response (H&E stain, 20× magnification). (D) Spongiotic dermatitis with liquefaction degeneration of basal cells, characteristic of Stevens–Johnson syndrome (H&E stain, 20× magnification). (E) Reticulated basket-weave stratum corneum with spongiotic edema of the stratum spinosum, indicative of chronic dermatitis (H&E stain, 20× magnification). (F) Mild vacuolization and degeneration of basal cells with a sparse inflammatory cell infiltrate, suggesting a less severe drug rash (H&E stain, 20× magnification).

Skin histopathology: epidermal hyperkeratosis with focal hyperkeratosis, scattered necrotic keratinocytes in the stratum spinosum, vacuolar degeneration in the basal layer, infiltration of lymphocytes, histiocytes and scattered eosinophils in the superficial dermal layer around blood vessels (Fig. 2A, B).

Treatment: Methylprednisolone injection 80 mg bid intravenously (reduce the amount according to the rash). Plasma replacement 2000 mL/time with propecia 5 g/d alternately. Active anti-infection based on pathogenesis, nutritional support, and stabilization of the internal environment. According to the improvement of the rash, the dose of methylprednisolone was adjusted, and methylprednisolone was reduced to 24 mg/d when discharged.

### 2.2. Case 2

A 75-year-old male patient presented with erythema, flakiness, and pruritus affecting his entire body for a duration exceeding 2 months. The patient was diagnosed with prostate cancer 7 years prior through physical examination, which was managed with oral abiraterone until its discontinuation in 2015 due to the onset of myasthenia gravis, at which point his creatine kinase levels exceeded 20,000; these levels improved following the cessation of the medication. In January 2022, the patient was prescribed 240 mg/d of oral apalutamide. By the end of March 2022, he developed erythema and pruritus localized to both anterior shins, which subsequently disseminated across his body. Despite the cessation of abatacept, the rash persisted and was characterized by significant desquamation (Fig. 1C, D).

Past history: history of hypertension and arrhythmia.

Specialized examination: generalized erythema and papules, fused into patches, partially hypertrophic moss-like, with flaking, symmetrically distributed, especially on the forehead and anterior shin.

Skin histopathology: epidermal focal hyperkeratosis, hypertrophy of the stratum spinosum, focal mild spongiotic edema, a small number of lymphocytes, histiocytes and eosinophils infiltrate around small blood vessels in the superficial dermis. It may be consistent with “chronic dermatitis” (Fig. 2C, D).

Treatment: Duplizumab 600 mg subcutaneous injection, 300 mg subcutaneous injection after 2 weeks, every 2 weeks.

### 2.3. Case 3

A 65-year-old male presented with widespread erythematous papules and mucous membrane eruptions persisting for 23 days. The patient commenced apalutamide treatment at a dosage of 240 mg/d at the end of March 2022, following a diagnosis of prostate cancer with bone metastasis. On May 3, 2022, the patient exhibited erythema and papules on both upper limbs, accompanied by pruritus and subjective reports of muscular pain in the limbs, without the presence of fever (Fig. 1E, F). Two days later, he experienced redness and swelling of both eyelids, along with chest tightness and discomfort, as well as pain during swallowing, which adversely affected his ability to eat and was associated with a loss of taste. The patient received Medrol at a dosage of 12 mg from an external facility. Subsequently, he was prescribed Medrol 12 mg 3 times daily, cetirizine hydrochloride, and a topical corticosteroid. After 3 days, the patient independently reduced the dosage, which resulted in the development of oral mucosal erosion. Three days prior to this report, the dosage was increased to Medrol 16 mg 3 times daily, leading to a reduction in the new rash and an improvement in the mucosal erosion.

Past history: history of allergic rhinitis and childhood asthma.

Specialized examination: dark erythematous spots and papules on the head, face, trunk and limbs, fused into a large number of mounts with desquamation, perioral erythema, chapped and crusted lips, mild vesicles at the corners of the mouth, negative Nystrom sign.

Skin histopathology: reticulated basket-like stratum corneum, spongy edema of the stratum spinosum, liquefaction and degeneration of some basal cells, infiltration of a small number of lymphocytes and histiocytes around small blood vessels in the superficial dermis (Fig. 2E, F).

Treatment: methylprednisolone injection 32 mg qd sedation, with oral antihistamine drugs and topical glucocorticoid ointment.

### 2.4. Case 4

A 69-year-old male patient, diagnosed with prostate cancer on November 15, 2021, commenced treatment with apalutamide at a dosage of 240 mg/d on November 29, 2021. One week following the initiation of therapy, the patient began to exhibit multiple erythematous lesions on both lower extremities, which progressively disseminated throughout the body. The administration of apalutamide was discontinued on December 28, 2021, and the patient was subsequently prescribed prednisone tablets at a dosage of 2 tablets twice daily. Despite this intervention, there was minimal improvement in the patient’s symptoms, and the rash became generalized, accompanied by widespread edema, without the presence of persistent fever. The patient’s medical history includes hypertension, for which he regularly takes Dyvan, as well as a cerebral infarction that occurred 6 years prior, for which he has been on long-term treatment with Byretol and Lipitor (Fig. 1G, H).

Specialized examination: flaky edematous erythema of the head, face, trunk and limbs, scattered erosions of the oral mucosa, conjunctival vulvar mucosa (‐).

Treatment: methylprednisolone 40 mg qd intravenous, propecia 20 g qd * 3 days, methylprednisolone according to the improvement of the rash gradually reduced. February 15, 2022, the patient self-administered apalutamide again, 3 days later the rash appeared again, facial erythema, accompanied by bilateral hand and foot nail dystrophy. The generalized rash subsided on its own 1 week after discontinuation of the drug, with no improvement in nail symptoms.

## 3. Discussion

Apalutamide-associated skin adverse events are increasingly recognized with the expanded use of this novel next-generation oral antiandrogen receptor targeting agent for castration-resistant prostate cancer.^[3]^ Our case series highlights the diverse clinical presentations of these AEs, ranging from eczema to severe drug rashes such as toxic epidermal necrolysis and Stevens–Johnson syndrome (SJS). The incubation periods varied from 1 week to 2 months, indicating that clinicians should remain vigilant for skin manifestations throughout the initial months of treatment (Table 1). The histopathological findings, including epidermal hyperkeratosis, focal hyperkeratosis, and infiltration of inflammatory cells, further support the diagnosis and provide insights into the underlying pathophysiology.^[3]^

**Table 1:** Clinical and laboratory characteristics of 4 patients with apalutamide-associated skin adverse events.

Cases	incubation/latent period	Rash type	WBC	EOS	CRP	LDH	Lymph nodes enlargement	Fever	Others
Case 1	2 months	TEN	7.79	0.00	84	550	+	+	Liver damage
Case 2	2 months	Eczema	6.8	1.9	2.80	357	‐	-	‐
Case 3	1 month	Eczema	4.9	0.00	6.4	366	‐	‐	‐
Case 4	1 week	SJS	5.55	2.72	10	783	‐	‐	Nail damage

CRP = C-reactive protein, EOS = eosinophil count, LDH = lactate dehydrogenase, SJS = Stevens–Johnson syndrome, TEN = toxic epidermal necrolysis, WBC = white blood cell count.

The management of apalutamide-associated skin AEs is crucial for maintaining patients’ quality of life and ensuring the continuation of cancer treatment when possible. Our cases demonstrate that discontinuation of apalutamide is often necessary to control the progression of skin rashes.^[4]^ Systemic glucocorticoid therapy was effective in 3 of our cases, highlighting its role in managing severe skin reactions. In one case, Duplizumab was used successfully, suggesting that biologic agents may offer an alternative treatment option for refractory cases. The reemergence of rash upon readministration of apalutamide in 1 of our patients underscores the importance of dose adjustment or discontinuation to prevent recurrent severe skin reactions.

A multidisciplinary approach involving oncologists and dermatologists is essential for optimizing the management of apalutamide-associated skin AEs. Oncologists play a critical role in weighing the benefits of continued cancer treatment against the risks of severe skin reactions, while dermatologists provide expertise in diagnosing and treating complex skin conditions. This collaborative approach ensures that patients receive comprehensive care tailored to their individual needs, balancing the goals of cancer therapy with the need to mitigate adverse effects on skin health.

Our findings emphasize the need for further research to better understand the pathogenesis of apalutamide-associated skin AEs. The dose-dependent nature of these reactions suggests that careful monitoring and adjustment of drug doses may help mitigate the risk of severe skin complications. Additionally, the identification of predictive biomarkers could aid in early detection and prevention of these AEs. Future studies should focus on large cohorts of patients treated with apalutamide to determine the frequency, time to onset, and clinicopathologic features of skin AEs more precisely. This information will be invaluable for developing evidence-based guidelines for the management of these challenging adverse events.

Our case series provides valuable insights into the clinical presentation, diagnosis, and management of apalutamide-associated skin AEs. A multidisciplinary approach and prompt intervention are key to optimizing patient outcomes and maintaining quality of life. Further research is needed to enhance our understanding of the underlying mechanisms and to develop more effective management strategies for these AEs.

## 4. Conclusion

4 cases of drug-related reactions, including 2 cases of eczema, 2 cases of severe drug rash, manifested as toxic epidermal necrolysis and SJS. 1 case of SJS rash subsided, and drug reactions rapidly appeared after readministration of the drug after discontinuation of drug. Combined with the history of drug use and histopathological changes in the skin, the diagnosis was clear. Case 1 with low eosinophils cells were considered to be relevant after systemic high-dose glucocorticoid therapy that was given in the outside hospital. After the appearance of drug rashes in the 4 patients, all of them discontinued the use of apalutamide, oral antihistamines and topical glucocorticoid ointment, and the rashes progressed, and systemic treatment with glucocorticoid was given in 3 cases, and Duplizumab was administered to 1 case, and only then did the symptoms gradually improve.

Apalutamide-associated skin AEs develop in a dose-dependent manner. Therefore the drug’s doses should be discontinued or reduced for controlling associated skin AEs. The time to ***apalutamide—discontinuation after the incidence of skin adverse events was positively correlated with the worsening of these events.

With expanded approval of apalutamide in castration sensitive and resistant prostate cancer, the impact of dermatologic adverse events (dAE) on dosing may increase. Understanding apalutamide-related dAE clinical presentation and management may help maintain quality of life and mitigate dose interruptions. We determine dAE frequency and time to onset and characterize clinicopathologic features in a large cohort of prostate cancer patients treated with apalutamide and recommend management strategies based on our multidisciplinary (oncologist–dermatologist) experience.

## Author contributions

**Conceptualization:** Yuanyuan Li, Yi Cao, Lei Wei, Jingyi Zhao, Maocan Tao, Xiaoqing Zhao.

**Data curation:** Yuanyuan Li, Yi Cao, Lei Wei, Jingyi Zhao, Maocan Tao, Xiaoqing Zhao.

**Formal analysis:** Yuanyuan Li, Yi Cao, Lei Wei, Jingyi Zhao, Maocan Tao, Xiaoqing Zhao.

**Funding acquisition:** Yuanyuan Li, Yi Cao, Lei Wei, Jingyi Zhao, Maocan Tao, Xiaoqing Zhao.

**Investigation:** Yuanyuan Li, Yi Cao, Lei Wei, Jingyi Zhao, Maocan Tao, Xiaoqing Zhao.

**Methodology:** Yuanyuan Li, Yi Cao, Lei Wei, Jingyi Zhao, Maocan Tao, Xiaoqing Zhao.

**Project administration:** Yuanyuan Li, Yi Cao, Lei Wei, Jingyi Zhao, Maocan Tao, Xiaoqing Zhao.

**Resources:** Yuanyuan Li, Yi Cao, Lei Wei, Jingyi Zhao, Maocan Tao, Xiaoqing Zhao.

**Software:** Yuanyuan Li, Yi Cao, Lei Wei, Jingyi Zhao, Maocan Tao, Xiaoqing Zhao.

**Supervision:** Yuanyuan Li, Yi Cao, Lei Wei, Jingyi Zhao, Maocan Tao, Xiaoqing Zhao.

**Validation:** Yuanyuan Li, Yi Cao, Lei Wei, Jingyi Zhao, Maocan Tao, Xiaoqing Zhao.

**Visualization:** Yuanyuan Li, Yi Cao, Lei Wei, Jingyi Zhao, Maocan Tao, Xiaoqing Zhao.

**Writing – original draft:** Yuanyuan Li, Yi Cao, Lei Wei, Jingyi Zhao, Maocan Tao, Xiaoqing Zhao.

**Writing – review & editing:** Yuanyuan Li, Yi Cao, Lei Wei, Jingyi Zhao, Maocan Tao, Xiaoqing Zhao.
